# Flavin Adenine Dinucleotide (FAD) Pegylated (PEG)-Complexes: Proof of Concept (PoC) of theranostic tool on a Murine Breast Cancer Model

**DOI:** 10.7150/ntno.63496

**Published:** 2022-01-01

**Authors:** Celia Arib, Hui Liu, Qiqian Liu, Anne-Marie Cieutat, Didier Paleni, Xiaowu Li, Jolanda Spadavecchia

**Affiliations:** 1 CNRS, UMR 7244, NBD-CSPBAT, Laboratoire de Chimie, Structures et Propriétés de Biomatériaux et d'Agents Thérapeutiques Université Sorbonne Paris Nord, campus Bobigny, France.; 2 Department of Hepatobiliary Surgery, Guangdong Provincial Key Laboratory of Regional Immunity and Diseases & Carson International Cancer Center, Shenzhen University General Hospital & Shenzhen University Clinical Medical Academy Center, Shenzhen University, Shenzhen, China.; 3 BioEVEN start-up, 75 rue de Lourmel 75015 Paris, France.

## Abstract

Flavin adenine dinucleotide (FAD) plays a key role in an extensive range of cellular oxidation-reduction reactions, which is engaged in metabolic pathways. The purpose of this study was to realize pegylated flavins formulation, named FAD and FAD-PEG diacid complex as theranostic tool in cancer therapy. For this objective, a murine breast cancer model, which was induced by mouse-derived4T1 breast cancer cells was studied to assess the therapeutic efficacy of FAD (named NP1) and FAD-PEG diacid complex (named NP2). The cytokines were monitored to evaluate the serum inflammatory factors to develop the blood cell content of different groups of nude mice. The experimental model shows that an intravenous injection of FAD (NP1) can significantly reduce tumour volume, tumour index and thymus index, and decrease neutrophils (NE), monocytes (MO), eosinophils (EO), and basophils (BA). At the same time, the content of IL-1α, IL-12P70, TNF α, IL-1β and IL-6 was significantly reduced, and the content of IL-10 was significantly increased. These results provide the proof-of-concept for FAD as a smart adjuvant for cancer therapy and encourages their further development in the field of Nanomedicine.

## Introduction

Breast cancer is the most commonly occurring cancer in women and overall the second most common cancer in the world 1,2. There are different limitations to conventional therapy for breast cancer 3. Nanocarriers have the potential to provide a high drug loading capacity, stability, exceptional tolerability, drug deterioration, decrease multidrug resistance, controlled release and encouraged delivery of anticancer drugs 4. Consequently, therapeutics and theranostics drug delivery nanocarrier systems have significant benefits over common treatment methods 5. Nevertheless, there are many challenges needed to be resolved in drug delivery systems, such as undue accumulation of the carriers in the liver, poor therapeutic efficacy on the cancer cells and the barriers near the tumour areas or in the vasculature area; all of these were realized as the obstacles to penetrate into cancer cells and are needed to be resolved. Flavin adenine dinucleotide (FAD) plays a part in various metabolic reactions where the biological function is naturally connected to its structure conformation change 6. Its main role is to be a cofactor, which is required for the activity of several flavoproteins, and is important for electron transport pathways in many living systems 7,8. FAD synthase is the last enzyme in the metabolic pathway producing FAD, a protein confined in both cytosol and in mitochondria 9,10 and it regulates different processes for cell life and death, resulting in ROS production, antioxidant defence, protein folding, and chromatin remodelling 10. Recent studies show that the modifications in coenzyme levels have been recorded in several cancers 11. Coenzymes take part in regulating enzyme activity to perform disparate biochemical reactions 12,13. Mutations in metabolic enzymes obstruct, typically, biochemical reactions leading to many disorders 14-16. Recently J. Spadavecchia and D. Paleni, conceived for the first time, the role of FAD cofactor as nanodevice in cancer therapy 17. The first aim of this present study, was the evolution of novel adjuvant in chemotherapy by using FAD alone or complexed to pegylated chains to obtain hybrid complex (FAD PEG-Diacid) and compared with a common chemotherapeutic (5 Fluro Uracil; 5-FU) in the therapy of cancer. For this purpose, a mouse breast cancer model induced by mouse-derived 4T1 breast cancer cells, was used to evaluate the therapeutic efficacy of FAD (named NP1) and FAD-PEG diacid complex (named NP2). We believe that this study is important for planning optimal chemotherapeutic systems in order to comprehend the mechanisms of poor effects of 5-FU.

## Materials and Methods

### Methods

To evaluate the therapeutic effects of FAD (NP1) and FAD/PEG complex (NP2) in the 4T1 cells induced breast cancer model of mouse. Animals were divided into a sham group, 0.9% NaCl injection group, PEG group, NP1 solution intravenous injection group, NP2 solution intravenous injection group, NP1 solution oral administration group, NP2 solution oral administration group, and positive control 5-fluorouracil group, a total of 8 groups. An injection of 4T1 cells containing matrigel was administered into the second pair of breast pads on the left side of the mouse (the total number of injected cells is 2×10^6^). When the tumour model is successfully built, 100 μL of the drug is administered intravenously and orally, twice a day (Fixed at 9:00 am and 3:00 pm every day, 6 hours apart), 2 days a week (once every 3 days), for 3 weeks. The blood was collected after the animals were anesthetized by ketamine/xylazine. The tumours were removed, weighed, and the percentage of inhibition of the drug was calculated with a negative control. The liver, kidney, spleen, and thymus were dissected to calculate the ratio (index) to weight. Furthermore, whole blood count was detected using a haemocytometer before 1 week of 4T1 cell injection and before the first drug administration (DIV 8), 1 week (DIV 15), 2 weeks (DIV 22) and 3 weeks (DIV 29) number of cells. Flow cytometry was used to measure serum cytokines before 4T1 cells (DIV 0), 1 weeks after 4T1 cells were injected (DIV 8), 2 weeks after DIV15, 3 weeks (DIV 22), and 4 weeks (DIV 29).

### Materials

The Flavin Adenine Nucleotide, Dicarboxylic Poly Ethylene Glycol (PEG)-600, cell culture media, fetal bovine serum (FBS), and penicillin-streptomycin were obtained from Thermo Fisher Scientific (Waltham, MA, USA). The 5-fluorouracil (5-FU) was purchased from Shanghai Xudong Haipu Pharmaceutical Co., Ltd. (Shanghai, People's Republic of China).

The Bio Legend LEGENDplex^TM^ multiplex bead-based assay kit was from BioLegend (San Diego, CA, USA). The Matrigel^®^ Basement Membrane Matrix was from Corning (Tewksbury, MA, USA). HEMAVET950FS animal blood analyzer special reagents were from Drew Scientific, Inc., USA. Isoflurane was obtained from RWD Life Science Co., Ltd. (Shenzhen, People's Republic of China).All other reagents were either obtained from Sigma-Aldrich (St. Louis, MO, USA) or noted otherwise.

### Instruments

Animal Weight Balance (Mettler - Toledo instruments (Shanghai) Co., LTD*,* Serial number: PL3001-s), Drug Weighing Balance Serial number: AL204 Electronic Balance Mettler-Toledo instruments (Shanghai) Co., LTD. HEMAVET 950 Animal Blood Analyzer (Drew Scientific, Inc., USA- Model: HEMAVET 950FS), Automatic biochemical analyzer (Hitachi-Model: 7100), BD Accuri C6 Flowcytometer (BD company), High Resolution Small Animal Ultrasound Imaging System (Visual Sonics-Model: Vevo2100, probe MS400, frequency 30 MHz), Inductively Coupled Plasma Mass Spectrometer (ICAP-Q) (American Thermo Company).DB-3EFS type hot plate (Tianjin Gongxing Laboratory Instrument Co., Ltd), Milli-Q ultrapure water treatment system (American Millipore Company).

### Synthesis Procedures of FAD-PEG Diacid Complex (NP2)

The synthesis of FAD-PEG Diacid Complex (NP2) was described previously 18.

### Physical-Chemical characterization

All characterizations were carried out in triplicate determinations as described previously [4^_^18].

### Mice Tests

*In vivo* tests were performed using male nude mice BALB/c mice, Grade: SPF, age 5 weeks. Animals were randomly divided into eight groups: the sham group (n=10), the 0.9% NaCl injection group (n=8), the PEG group (n=8), the NP1 solution intravenous group (n=8), the NP2 solution intravenous group (n=8), the NP1 solution oral administration group (n=8), the NP2 solution oral administration group (n=8) and the 5-fluorouracil group as positive control (n=7). Animal production license number: SCXK (Yue) 2018-0034, Guangdong Medical Laboratory Animal Center; animal certificate number: No. 44005800009527; No. 44005800009345; animal Use License number: SYXK (Yue) 2018-0001, Laboratory animal Center, Guangzhou University of Chinese Medicine. The technical procedure was described previously 18.

### Quarantine

All experiments and technical procedures were recorded and described as in previous paper 18.

### Grouping and Administration

After the tumour model of nude mice was built. 100 μL of different test drugs were intravenously and orally administered twice every day (injection at 9:00 am and at 15:00 pm). The drug was administered two days every week (dosing every three days) and for a total of 3 weeks.

### Detection Indicators

Observation of the general condition, body weight and organ index of nude mice. The general situation of the nude mouse includes the activity, mental state, skin color, diet, water intake and urine output. Body weight (BW) was weighed every 3 days. At the end of the experiment, the heart, liver, spleen, lung, kidney and tumour were separated and accurately weighed after the nude mice were sacrificed, and the heart, liver, spleen, lung, kidney and tumour index were calculated.

That is:


*Tissue weight (mg)/body weight (g) =Tissue weight/BW.*

### Organ index detection

Body weight (BW) was weighed every 3 days. At the end of the experiment, the heart, liver, spleen, lung, kidney and tumor were separated and accurately weighted after the nude mice were sacrificed. The heart, liver, spleen, lung, kidney and tumor index were calculated by using the following formula:


*Organ index (mg/g) = Tissue weight/BW.*

### Tumor volume assessment

The growth of tumors was monitored every 3 days by caliper measurement of tumor length (a) and width (b). Tumor volume was estimated by using the following formula:


Tumor volume (mm^3^) = (a*b^2^)/2.

The Table of results was reported in Supporting Information (**Table S1**).

### Whole blood cell count detection

Whole blood was measured as previously described 18.

### Multiplex bead-based assay for pro-inflammatory cytokine

Flow cytometry was used to detect serum cytokine levels as previously described 18.

### Sample preparation

250 μL of assay buffer was added to the standard to form a liquor of 10000 pg/mL, and left at room temperature for 10 minutes; 8 tubes were taken, numbered C1-C7, 75 μL of assay buffer added to each tube, and then 25 μL of standard stock solution was added to C1 tube. After vortexing, 25 μL was transferred to C2 tube. By analogy to C6, 75 μL was retained in each tube.

### Flow cytometry

Flow cytometry analysis was carried out as previously described 18.

### Statistical analysis

Statistical analysis was performed by GraphPad Prism 8.0 software as previously described 18.

## Results and Discussion

Recently we have proved the ability of FAD formulations (NP1; NP2) as adjuvant in chemotherapy against hepatocarcinome 18. In particular we blended NP1-NP2 with 5-FU in which electrostatic charges between the chemical groups of the drug and FAD formulations accomplished the final hybrid formulation. On the basis of these hypothesis, we believe that NP1-NP2 under complex form, after internalization in cancer cells, could improve the therapeutic efficacy, due to a double action on nuclei and mitochondria for blocking the binding of aminoacyl-tRNA to the mRNA-ribosome complex with evident variation in driven force and electrolytic condition. It has been suggested that the expression of Glucose 6 phosphate dehydrogenases (G6PD) and transketolase (TKT) are positively correlated to the decreased overall and relapse-free survival in breast cancer. The synergic action of FAD-5FU formulation inhibits Glucose 6 phosphate dehydrogenases 19,20 and dihydropyrimidine (DPD) 21 with a simultaneous reparation , and inhibition of resistance and improvement of anticancer activity. For this purpose, we conceived a lot of experiments in which NP2 was tested as pegylated complex and compared with control (FAD; i.e. NP1) and anti-cancer drug (5-Fluoro-Uracile [5-FU]).

### FAD (NP1) and FAD/PEG complexes (NP2) did not affect body weight changes in mice with breast cancer

**Figure 1:**
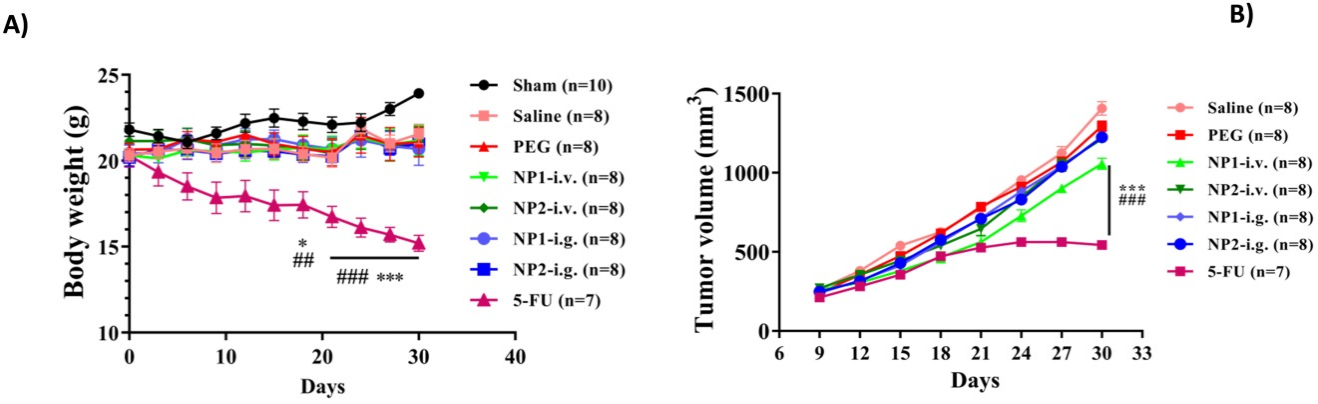
**A)** Effect of FAD on body weight change in breast mice with 4T1 cells injection. FAD was administered, b.i.d., orally or i.v. starting on 6 weeks of age continuously for a total of 4 weeks. Body weight was shown as Mean ± SEM. *p<0.05 and ***p<0.001 vs saline group mice, ##p<0.01 and ###p<0.001 vs PEG group mice. **B)** Effect of FAD on tumour volume in breast mice with 4T1 cells injection. Values were shown as Mean ± SEM. ***p< 0.001 vs saline group mice, ###p< 0.001 vs PEG group mice.

Breast cancer mice induced by 4T1 cells were used as a research model to evaluate the therapeutic effects of FAD(NP1) and FAD/PEG (NP2) complexes. The mice were weighed every 3 days. As shown in Fig. 1, the mice injected with 4T1 cells showed a different degree of decline compared with the sham group, the saline and PEG groups, the different administration methods of FAD (NP1) and FAD/PEG (NP2) complexes did not affect body weight of breast cancer model mice. However, the positive control drug 5-FU group, showed significant weight loss after 1 week of prophylactic administration, and had a statistical significance in DIV 18, DIV 21, DIV 24, DIV 27 and DIV 30 (p<0.05).

### FAD (NP1) intravenous injection significantly reduces tumour volume in mice

**Figure 2:**
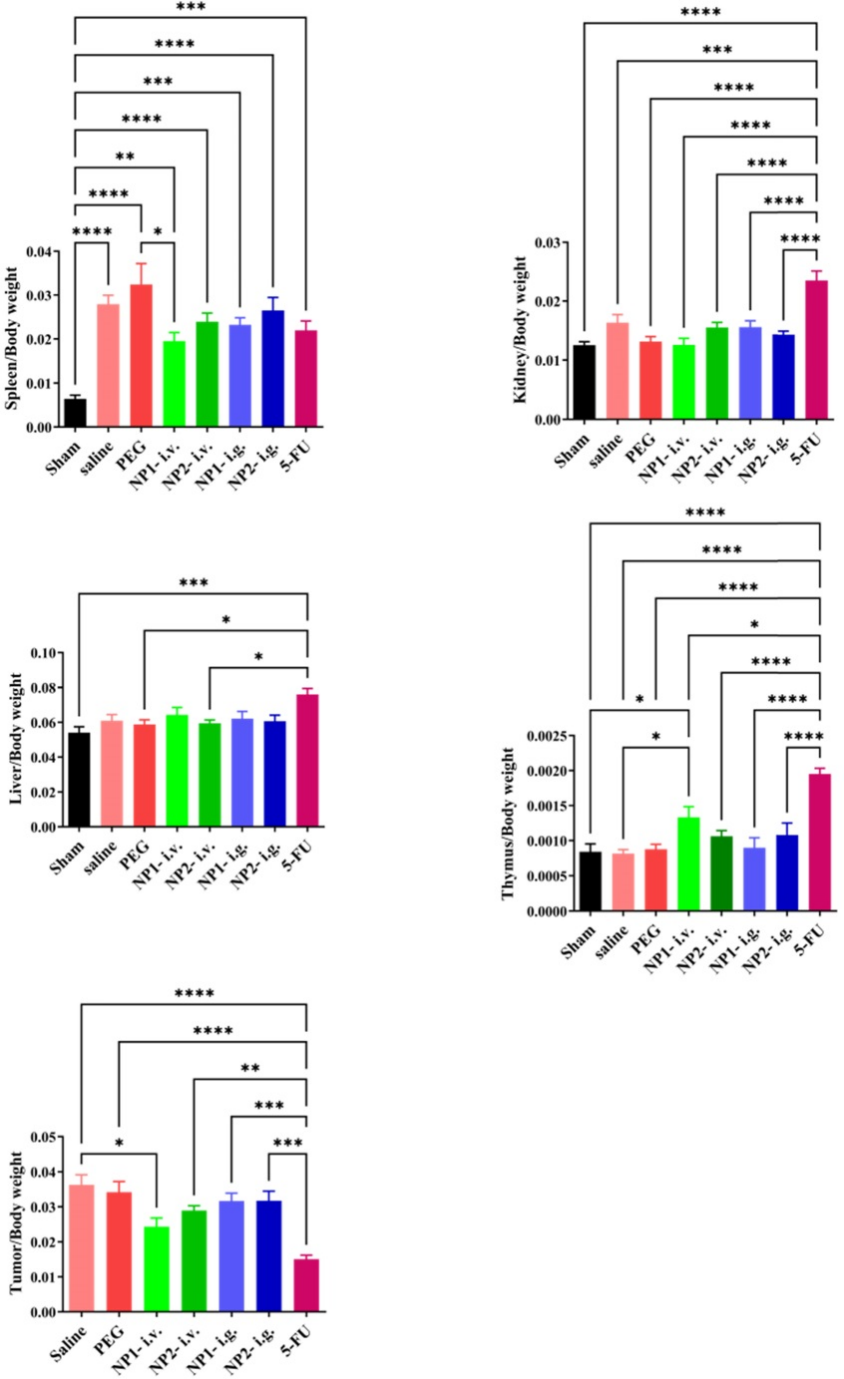
Effect of FAD (NP1) on Index (Spleen, Liver, Tumour, Kidney, Thymus) in breast mice with 4T1 cells injection. Values were shown as Mean ± SEM. n=10 for each group except sham group (n=14). &&&p<0.001 vs sham group mice, *p<0.05 and ***p<0.001 vs saline group mice, #p<0.05 and ###p<0.001 vs PEG group mice.

The tumour length and width of mice were measured every 3 days. As shown in Fig. 2, the volume of the 4T1 cell injection group gradually increased, and the NP1 and NP2 intravenous injection groups could delay the tumour volume increase. At the end of the experiment, compared with the first tumour volume, the tumour volume increased by 5.34 times, and the PEG group increased by 5.37 times. However, the tumour volume of the NP1 intravenous group and the NP2 intravenous group increased by 4.07 times and 4.50 times, respectively, which was significantly statistically different from the saline group and the PEG group (p<0.001). The tumour volume of mice in the 5-FU group was significantly smaller than that in the saline group and the PEG group, which had significant statistical significance (p<0.001).

### FAD intravenous injection significantly reduces tumour and spleen size in mice

At the end of the experiment, mouse tumours and spleen tissues were isolated and photographed. As shown in **Figure S1 in Supporting Information**, the tumour volume of mice in the NP1 and NP2 intravenous injection groups were smaller than that in the saline and PEG groups.

### FAD intravenous injection significantly reduces tumour index and spleen index

At the end of the experiment, the mice in each group were dissected to separate the spleen, kidney, liver, tumour and thymus, and the weight ratio of the organ to the corresponding mice was calculated. As shown in Fig. 2, the spleen index of the saline and PEG groups was significantly increased compared with the sham group (p<0.001). Compared with the PEG group, the NP1 intravenous injection group significantly reduced the spleen index (p<0.05). Compared with the saline group, the NP1 intravenous injection group can significantly increase the thymus index. Compared with the saline and PEG groups, the 5-FU group can significantly increase the thymus index (p <0.001). However, the kidney index and liver index in different groups there were no statistically significant differences. Compared with the saline group, the NP1 intravenous injection group can reduce the tumour index (p<0.05). Compared with the saline and PEG groups, the 5-FU group can also significantly reduce the tumour index (p<0.001). This behavior is probably due to specific chemical affinity of FAD conjugate to 5-FU, which induce a different steric arrangement of coenzyme and a consequent better therapeutic effect.

### FAD intravenous injection reduces NE, MO, EO, and BA numbers

**Figure 3:**
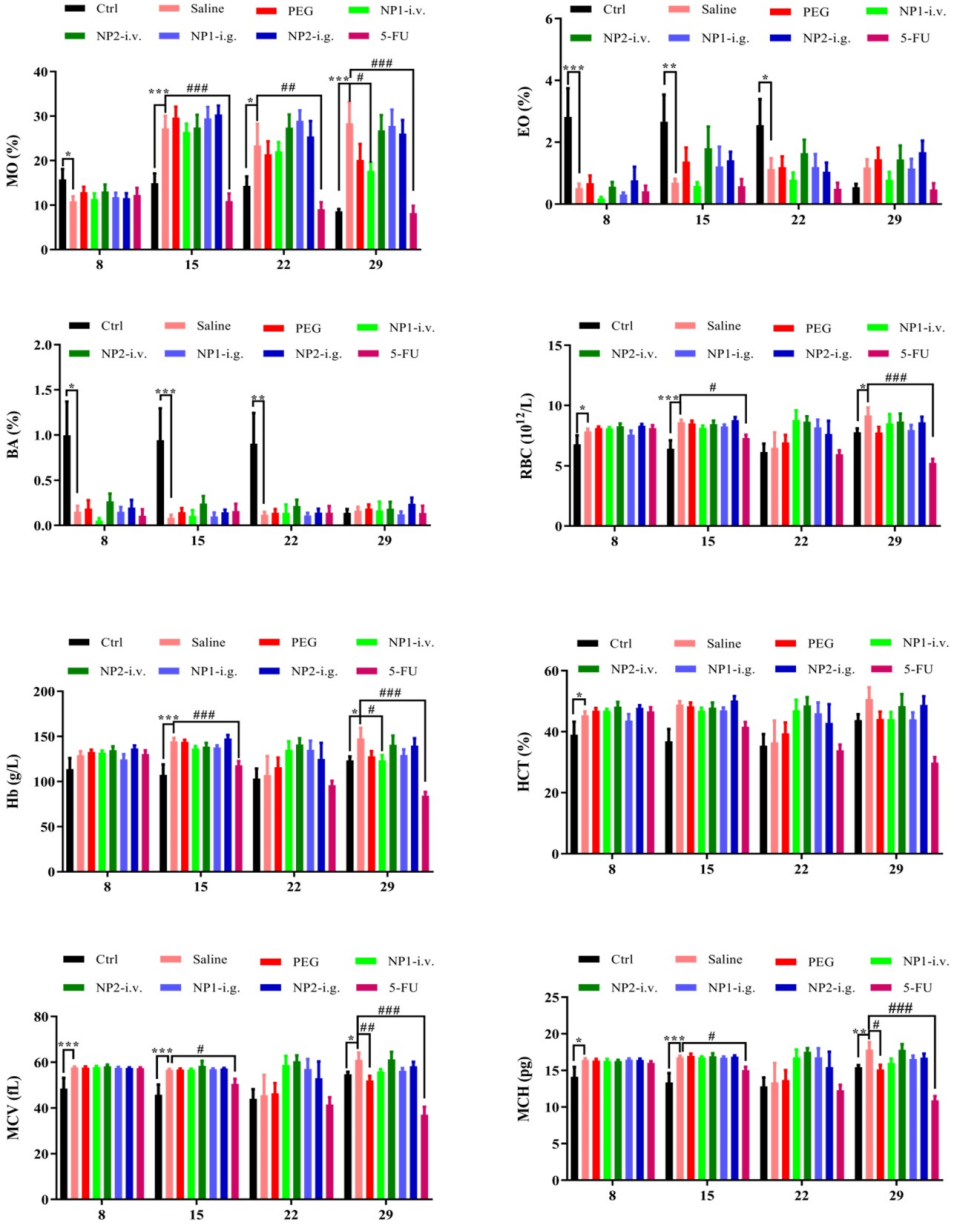

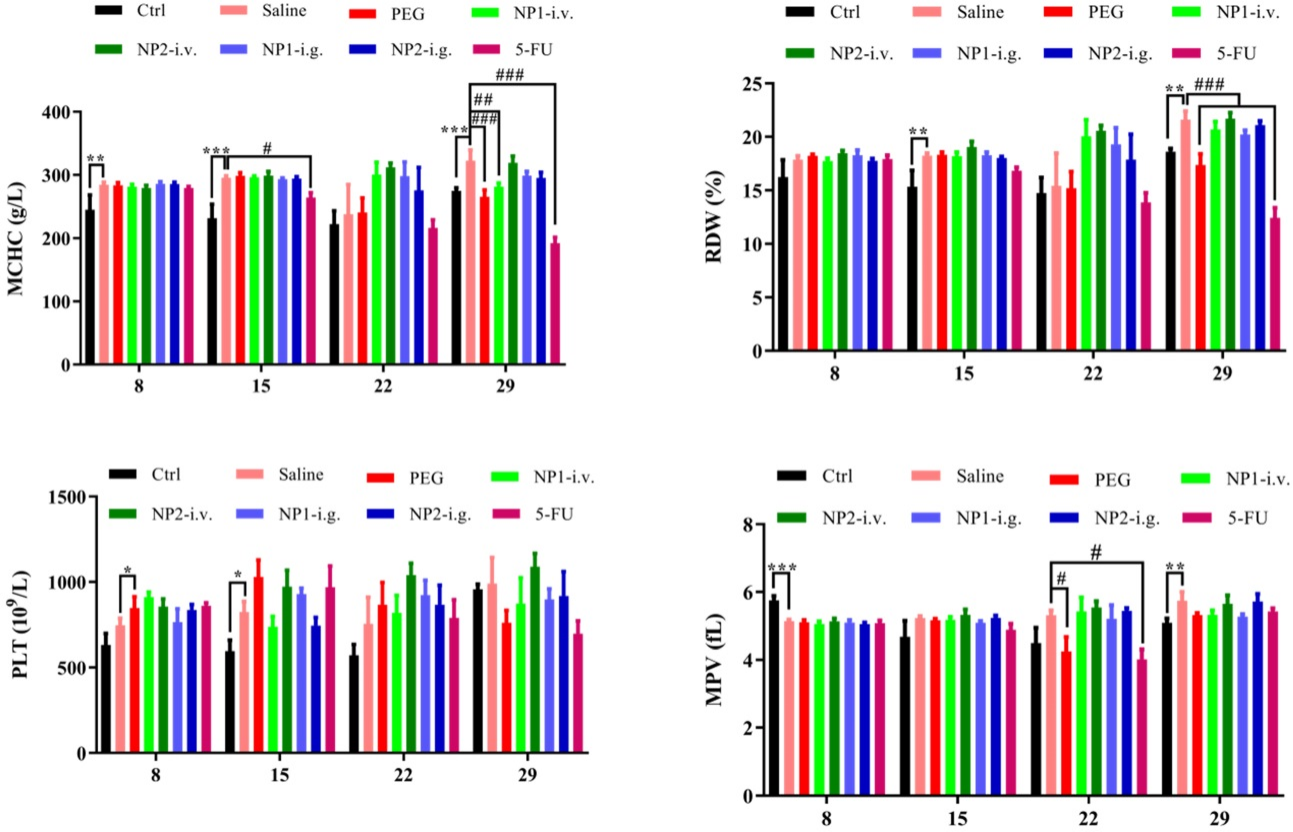
Effect of FAD on whole blood cell count in breast mice with 4T1 cells injection.

Whole blood was measured with a blood cell counter before the first week of drug administration and the injection of 4T1 cells (DIV 8), the second week after administration (DIV 15), the third week after administration (DIV 22), and the fourth week (DIV 29). As shown in Fig. 3, the number of NE, MO, EO, and BA in the NP 1 intravenous group has been reduced after 4 weeks of administration. Meanwhile, all indexes except the MPV index has been reduced in the 5-FU group. Contrarily to our previous study 18 in NP1 formulation, the steric arrangement of *free* FAD molecules, allows a better chemical interaction with 5-FU that permit a boost enhancement of blood cells that provoke inhibition of Glucose 6 phosphate dehydrogenases (G6PD) 20.

### Effects of FAD (NP1) and FAD/PEG (NP2) complexes on cytokines expression

Cytokines and chemokines are prompt proteins generated from various types of cells that regulate immune responses 22. There are two immune responses for cytokine production: 1) Antigen presenting cells (APCs) take up antigens, process them, and consequently follow them to T-lymphocytes to cause cytokines, 2) APCs, such as monocytes, are activated to generate cytokines by pattern recognition receptors that detect an extraneous pathogen 23. Cytokines mostly produced by APCs include multiple interleukin (IL) and tumour necrosis factor (TNF) molecules 24. Many clinical studies have demonstrated that inoculating a standard dose of chemotherapeutic drugs causes an enhancement in cytokine levels for a kind of cytokines (TNF-a, IL-6, IL-8, IL-10 25, and monocyte chemotactic protein-1 (MCP-1) 26. The lack to elucidate an injury can build extreme immune cell infiltration and lead to resolute cytokine production.

Previously we have demonstrated that the combination within NP2 (FAD-PEG Diacid Complex) and 5-FU have the potential of anti-inflammatory agents as they significantly reduced the serum levels of IL-6, TNF-α and IL-12 P70 probably due to anti-inflammatory properties of FAD 18.

**Figure 4:**
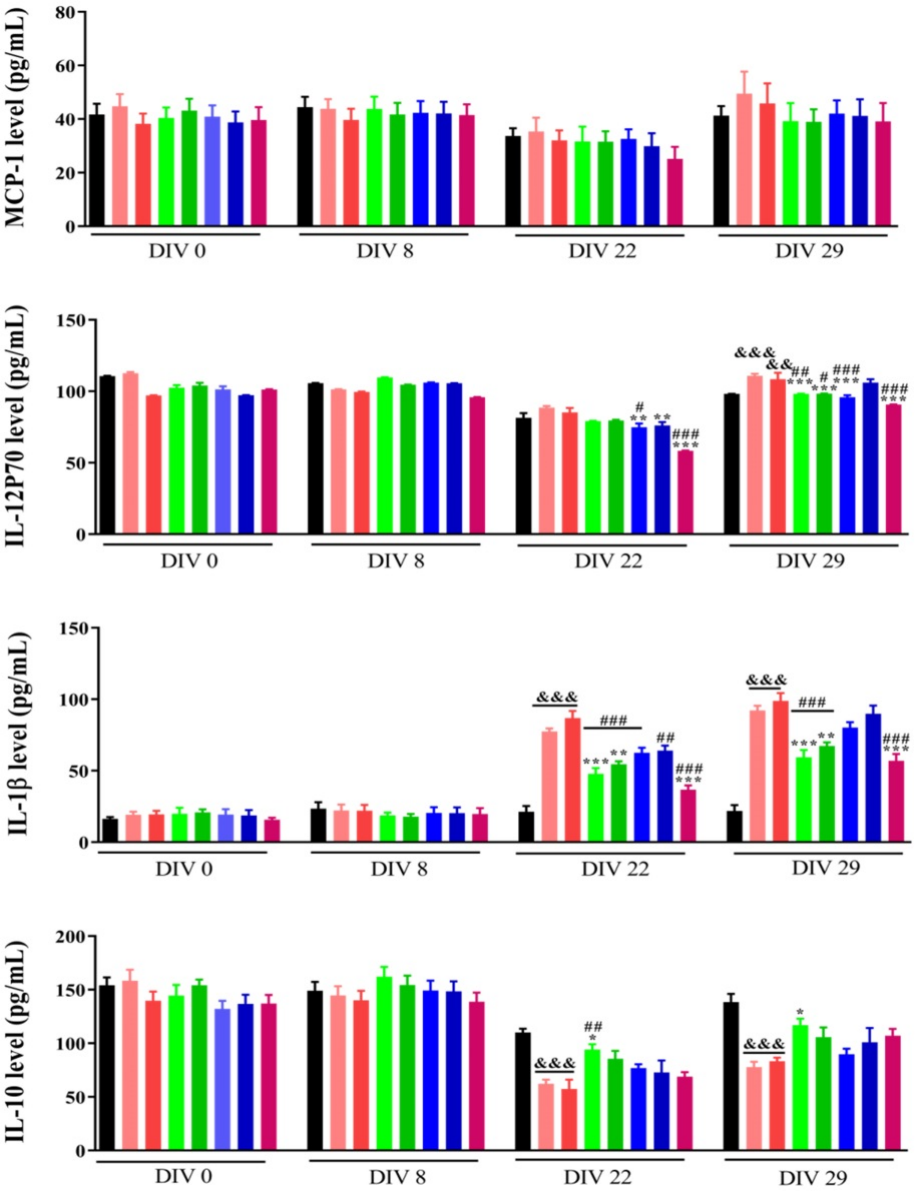

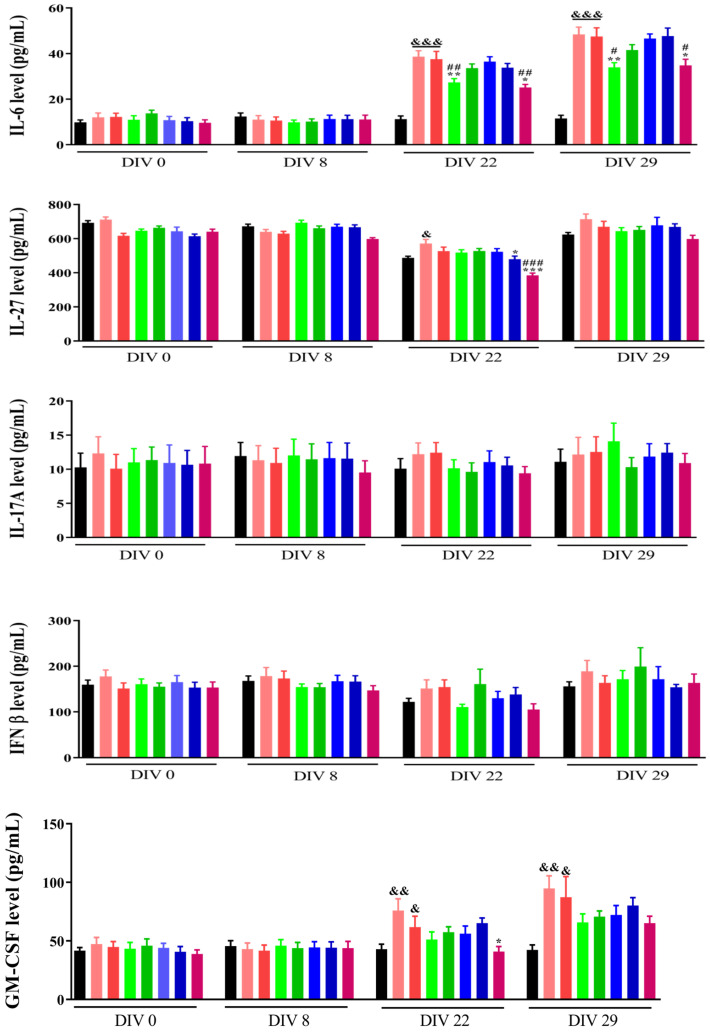
Effects of FAD on cytokine in breast mice with 4T1 cells injection. Values were shown as Mean± SEM. &p< 0.05, &&p< 0.01 and &&&p< 0.001 vs sham group mice, *p< 0.05, **p< 0.01 and ***p< 0.001 vs saline group mice, #p< 0.05, ##p< 0.01 and ###p< 0.001 vs PEG group mice.

Herein, flow cytometry was used to measure cytokines levels in serum before injecting 4T1 cells (DIV 0), 1 week (DIV 8), 2 weeks (DIV 22), and 3 weeks (DIV 29). As shown in Fig. 4, the levels of IL-23, MCP-1, IL-17A and IFN β were not different between the different groups at the corresponding time points. After 2 and 3 weeks of administration, the levels of IL-1α, GMCSF, and IL-6 were significantly increased (p<0.01), but the FAD (NP1) and FAD/PEG (NP2) complexes and the 5-FU group did not significantly affect IL-1α, GMCSF and IL-6 levels compared with the sham group. After 3 weeks of administration, compared with the sham group, the levels of IFN γ, TNF α, IL-12 P70, IL-1β and IL-27 were significantly increased, compared with the saline and PEG groups, FAD (NP1) and FAD/PEG (NP2) complexes and did not significantly improve the changes in the levels of IFN γ, TNF α, IL-1β, and IL-27. But the FAD intravenous injection group could significantly improve the changes of IL-12 P70 (p<0.01), and 5-FU could be different significantly reduced the levels of IFN γ, TNF α, IL-12 P70, IL-1β, and IL-27 (p<0.01). 4T1 cells were injected 1 week before the first drug administration (DIV 8), compared to sham group, the NP1-iv, NP 2-iv, and NP 1-ig groups significantly reduced the IL-10 levels (p <0.05) that plays a central role in regulating homeostasis resolving inflammation during acute infections or tissue injury at both a local and systemic level.

On the basis of these findings, we can suggest that NP1 decrease the level of pro-inflammatory cytokines and the synergic combination between NP1 and 5-FU strongly improve immune system effects 18.

## Conclusions

In this paper, we have analysed the importance of flavins coenzymes as a biocompatible formulation in a mouse model of breast cancer metabolism, as an adjuvant, and as an anticancer drug. Contrary to our previous paper 18 of the effect of FAD (NP1) and FAD/PEG (NP2), where the complexes have a booster effect on a murine model of liver cancer, on our murine model of breast cancer only intravenous injection of FAD can significantly reduce tumour volume, tumour index and increase thymus index; reduce neutrophils (NE), monocytes (MO), eosinophils (EO), and basophils (BA). At the same time, the content of IL-1α, IL-12 P70, TNF α, IL-1β and IL-6 was significantly reduced, and the content of IL-10 was significantly increased like in the previous liver cancer model. The advanced antitumor efficacy of FAD (NP1) compared to 5-FU is displayed, not only in the repression of tumour growth, but similarly in the stimulation of immune system. The signal pathway of FAD acting on blood cells/immune cells/tumour cells and the analysis of tumour tissue or immune cells will be studied in genomics and proteomics. These results predict the wave for the development of an innovative theranostic platform, allowing the detection of protein-associated tumours and the simultaneous cancer treatment with an adjuvant that reduce secondary effect of chemotherapy and boost immunity system with a consequent good replication of healthy cells.

## Supplementary Material

Supplementary figure and table.

## Figures and Tables

**Table 1:** Experimental animals were randomly divided into 8 groups: Experimental conditions of FAD formulations (NP1; NP2) administration

Group	Dose drugs	Number of mice	Method of administration	Administration frequency
Sham	Saline	10	i.v.	B.i.d
Saline	Saline	8	i.v.	B.i.d
PEG	PEG	8	i.v.	B.i.d
NP 1-i.v.	10 mg/kg	8	i.v.	B.i.d
NP 2-i.v.	10 mg/kg	8	i.v.	B.i.d
NP 1-i.g.	10 mg/kg	8	i.g.	B.i.d
NP 2-i.g.	10 mg/kg	8	i.g.	B.i.d
5-FU	10 mg/kg	7	i.v.	Q.d
