# Supplementary material for: Flavin Adenine Dinucleotide (FAD) Pegylated (PEG)-Complexes: Proof of Concept (PoC) of theranostic tool on a Murine Breast Cancer Model
Source: Nanotheranostics. 2022 Jan 1;6(2):175–83. doi: 10.7150/ntno.63496 (PMC8671949; doi:10.7150/ntno.63496)
Supplement: Supplementary file 1 — Supplementary figure and table. [file ntnov06p0175s1.pdf]

-Supporting Information-

Flavin Adenine Dinucleotide (FAD) Pegylated (PEG)-Complexes: Proof of Concept (PoC) of theranostic tool on a Breast Cancer Model of Mouse.

Celia Arib[1‡], Hui Liu[2‡], Qiqian Liu[1], Anne-Marie Cieutat[1],Didier Paleni[3], Xiaowu Li[2*],

Jolanda Spadavecchia[1-2*]

[1] CNRS, UMR 7244, NBD-CSPBAT, Laboratoire de Chimie, Structures et Propriétés de Biomatériaux et d'Agents Thérapeutiques Université Sorbonne Paris Nord, campus Bobigny, France

[2] Department of Hepatobiliary Surgery, Guangdong Provincial Key Laboratory of Regional Immunity and Diseases & Carson International Cancer Center, Shenzhen University General Hospital & Shenzhen University Clinical Medical Academy Center, Shenzhen University, Shenzhen, China

[3]BioEVEN start-up, 75 rue de Lourmel 75015 Paris

Corresponding Author*: lixw1966@163.com; jolanda.spadavecchia@univ-paris13.fr

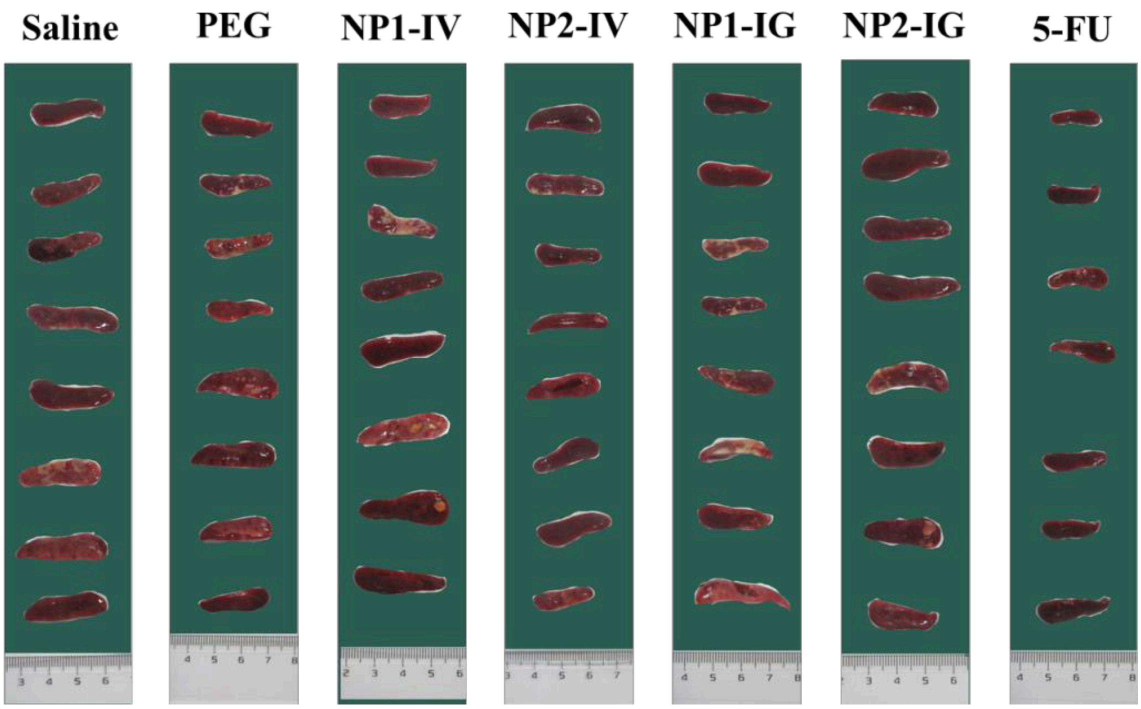

Fig.S1. Representative images of tumour and spleen.

Day	Saline	Saline	Saline	Saline	Saline	Saline	Saline	Saline	Saline	Saline	Saline	Saline	Saline	Saline
9	276,7967		283,3618	266,7478	233,7225		316,3398	245,7031	240,0083	245,3475				
12	339,1465		358,6089	394,9658	359,049		462,31	418,7039	355,0159	353,3665				
15	492,2683		485,5563	578,1248	522,2117		570,5258	626,8375	500,9282	538,295				
18	692,2766		567,7564	557,4733	536,5253		673,0278	715,6293	646,0342	619,1705				
21	786,8209		672,7914	712,234	791,5057		807,9778	859,8271	774,2031	777,4467				
24	887,8603		963,5739	932,09	1006,94		1024,264	1066,741	923,7141	833,5004				
27	1034,444		1388	1063,69	1110,142		1022,459	1170,574	1111,236	1097,671				
30	1279,398		1610,51	1329,87	1411,757		1233,355	1479,752	1440,273	1467,047				

Day	PEG	PEG	PEG	PEG	PEG	PEG	PEG	PEG	PEG	PEG	PEG	PEG	PEG	PEG
9	242,18	238,198	257,271	199,908	206,0142		253,9827	280,131	251,2019					
12	342,8768	436,978	334,43	322,24	266,176		386,0244	389,5666	369,3153					
15	562,8311	546,1827	398,834	368,19	373,2019		544,3638	487,075	509,8961					
18	652,9623	655,9958	612,08	582,246	497,1192		611,6132	610,7	710,6447					
21	734,326	866,4955	800,1	756,26	690,3936		728	845,0255	864,4518					
24	935,8522	923,4908	946,88	881,8262	829,8725		935,5117	952,4321	903,1501					
27	1034,983	1088,95	1006,351	910,1351	1154,901		1152,668	1180,616	1010,605					
30	1166,746	1364,298	1238,151	1229,151	1338,777		1457,688	1270,147	1289,115					

Day	NP1-i.v.	NP1-i.v.	NP1-i.v.	NP1-i.v.	NP1-i.v.	NP1-i.v.	NP1-i.v.	NP1-i.v.	NP1-i.v.	NP1-i.v.
9	215,3415	245,9084		281,3935	308,0262	277,2455	187,78	302,8009	257,8662	
12	311,9899	281,9923		355,0479	329,1733	325,5049	224,98	308,9135	310,6301	
15	421,3904	336,0473		389,5178	357,3786	412,7086	298,3645	403,8478	419,2814	
18	659,3805	455,204		426,9218	367,7599	528,9036	332,7537	441,3447	508,1341	
21	737,7408	519,3323		554,6812	491,8109	596,4918	387,8912	539,0228	665,7646	
24	843,8634	752,8367		704,9898	594,8221	710,469	581,4653	760,6437	875,7484	
27	985,1591	990,4699		880,745	795,767	915,2351	795,9154	892,9423	956,6212	
30	1051	1179,068		911,1276	1041,29	1106,574	974,1093	991,686	1195,178	

Table S1: Descriptive Table of all results regarding Tumor Volume Change
